# Hormonal profile and androgen receptor study in prepubertal girls with hypertrichosis

**DOI:** 10.1186/1687-9856-2014-6

**Published:** 2014-04-18

**Authors:** Maria Isabel Hernandez, Andrea Castro, Ketty Bacallao, Alejandra Avila, Aníbal Espinoza, Leon Trejo, Germán Iñiguez, Ethel Codner, Fernando Cassorla

**Affiliations:** 1 Institute of Maternal and Child Research (IDIMI), Faculty of Medicine, University of Chile, Santa Rosa 1234, 2nd floor IDIMI, Casilla, 226-3 Santiago, Chile; 2 Departments of Pediatrics, Hospital Clínico San Borja Arriarán, Santiago, Chile; 3 Department of Radiology, Hospital Clínico San Borja Arriarán, Santiago, Chile

**Keywords:** Hypertricosis, Prepubertal girls, Androgen receptor, Body hair, Androgen metabolism

## Abstract

**Background:**

Prepubertal hypertrichosis is a reportedly benign condition characterized by an excessive growth of vellous hair in non-androgen dependent areas of the body compared to the amount usually present in normal subjects of the same age, race and sex. Although this condition is usually considered idiopathic and regarded as benign, it may be very disturbing cosmetically, causing significant patient and parental anxiety.

**Method:**

We performed a hormonal and androgen receptor study in 42 prepubertal girls with hypertrichosis and 29 control girls from 2 to 8 years of age. Both groups underwent a determination of basal LH, FSH, 17OH progesterone, androstenedione, testosterone, estradiol and SHBG, abdominal ultrasound to assess ovarian morphology, and the number of androgen receptor CAG/GGC repeats in DNA obtained from peripheral leukocytes.

**Results:**

The hypertrichosis score was higher in the cases compared to controls. Serum gonadotropins and sex steroids were similar in both groups, but SHBG was significantly lower in the girls with hypertrichosis (71.1 ± 2.9 vs 81.9 ± 3.0 nmol/L, p < 0.02). The distribution of shorter, larger and total alleles was not statistically different between cases and controls. The combined analysis of CAG/GGC, however, showed a significantly higher prevalence of the most androgen-sensitive haplotypes (1–2: <22CAG + 17/17GGC- < 14CAG + 17/18GGC) in girls with hypertrichosis compared to controls.

**Conclusions:**

We conclude that girls with hypertrychosis exhibit AR(s) with enhanced sensitivity, which may facilitate the growth of their body hair.

## Introduction

Prepubertal hypertrichosis is a reportedly benign condition characterized by an excessive growth of vellous hair in non-androgen dependent areas of the body compared to the amount usually present in normal subjects of the same age, race and sex [1]. Characteristically, the hair in subjects with hypertrichosis usually grows on the trunk, arms and legs. This form of hypertrichosis was described by Barth et al. in 1988 [2], and differs from universal congenital hypertrichosis [3,4] and hypertrichosis laguginosa [5]. Although this condition is usually considered idiopathic and regarded as benign, it may be very disturbing cosmetically, causing significant patient and parental anxiety. In addition, hypertrichosis may be associated with metabolic or genetic disorders, and with the use of drugs [6-11].

Studies concerning the hormonal profile of subjects with hypertrichosis are relatively few and difficult to compare, due to the various definitions of this condition. In addition, the investigation of androgen metabolism has not shown significant differences between girls with hypertrichosis and controls [12,13].

The human androgen receptor (AR) is the main regulator of androgen signaling in the cell [14] and is encoded by the AR gene encoded in the X-chromosome [15]. The transactivator domain of the AR protein contains two polymorphic trinucleotide repeats segments that encode poly -glutamine (CAG)_n_ and –glycine (GGN)_n_ tracts within exon 1. There are considerable data linking the length of CAG repeats to androgen sensitivity *in vivo* as well as *in vitro,* that suggests an inverse relationship between length repeats and androgen receptor sensitivity [16]*.* In the case of GGC, AR with repeats number other than 17 GGC(23GGN), exhibit lower transactivation in response to androgens, suggesting a relationship between repeat lengths and AR function [16].

The aim of our study was to evaluate the clinical and hormonal characteristics of a large group of girls with hypertrichosis, and to assess the number of CAG and GGC repeats in their androgen receptor gene.

## Subjects and methods

### Subjects

A group of 42 prepubertal girls with hypertrichosis was recruited from our endocrine clinic, and matched by age with a group of 29 prepubertal control girls without hypertrichosis. The age of both groups of girls ranged between 2 to 8 years. Exclusion criteria were: 1) Any clinical sign of puberty according to Marshall and Tanner stages, ultrasonography or hormonal profile. 2) Chronic disease (including congenital adrenal hyperplasia) 3) Medication that could induce hypertrichosis or hyperandrogenism. 4) Low birth weight and 5) Maternal history of polycystic ovary syndrome.

### Study protocol

After an overnight fast, all girls were evaluated at the Institute of Maternal and Child Research of the University of Chile. The patients and control girls underwent a careful physical examination performed by a single pediatric endocrinologist (MIH), to determine their hypertrichosis score according to Gryngarten et al. [13]. As indicated by this author, a score above 7 was considered positive for hypertrichosis.

Height was measured using a Harpenden stadiometer (Holtain, UK). Weight was measured using a manual scale with a 10-g gradation (Seca; Quickmedical, Snoqualme,WA). All the measurements were expressed as SDS for chronological age.

We obtained a hand x-ray to determine the bone age according to the method of Greulich and Pyle [17]. This determination was performed by a single observer (AE), who was blinded to the condition of the girls. A transabdominal ultrasound was performed by a single ultrasonographist (AE) with a 5-MHz transducer in Sonoace 6000C equipment (Madison Co., Seoul, Korea). Ovarian volume was calculated using the simplified formula for a prolate ellipsoid [18] and we determined the number of follicles in each ovary.

Basal levels of androstenedione, FSH, LH, estradiol, testosterone, 17 hydroxyprogesterone (17OH Prog), SHBG, antimullerian hormone (AMH) and inhibin-B were obtained and measured in a fasting sample at 8:00 AM. SHBG and testosterone were used to calculate the free androgen index (FAI), as previously reported [19].

### Hormone assays

Serum testosterone, androstenedione, 17OH Prog, DHEA-S and estradiol were determined by competitive specific binding RIA, and serum LH, FSH, and SHBG were measured by immunoradiometric assays. All kits were supplied by Diagnostic System Laboratories (Webster, TX). Intra-assay coefficients of variation were 5.1% for testosterone, 3.2% for androstenedione, 4.2% for 17-OH Prog, 3.5% for DHEA-S, 4,1% for estradiol, 6.5% for LH, 3.6% for FSH, and 3.9% for SHBG. Interassay coefficients of variation were 6.4% for testosterone, 6.1% for androstenedione, 5.5% for 17-OH Prog, 5.1% for DHEA-S, 6.7% for estradiol, 7.6% for LH, 6.2% for FSH, and 6.9% for SHBG. Serum AMH was assayed using the AMH/MIS ELISA kit (Immunotech- Beckman, Marseilles, France). The AMH assay had a sensitivity of 0.7 pmol/L, and intra- and interassay coefficients of variation of 5.3% and 8.7% respectively. Serum inhibin B was measured using specific two-site ELISAs (Diagnostic Systems Labs, Webster TX). The assay sensitivity was 7 pg/mL, and intra- and inter-assay coefficients of variation were 4.8% and 7.1% respectively.

### Determination of CAG and GGC repeats

Genomic DNA was extracted from peripheral blood lymphocytes using a DNA blood Wizard kit. The AR exon 1 region encoding the polyglutamine repeat was amplified using PCR. To determine the length of the CAG and GGC repeats, we amplified the corresponding regions located on exon 1 of the AR gene (Genebank accession nº AL049564) using a pair of primers whose sequence has been previously reported [20,21]. One primer from each pair was marked with fluorescent dye (FAM or NED). Amplification was performed in a 15 μL reaction volume, containing 150 ng of DNA, 200 M of each deoxynucleotide triphosphate (Invitrogen, USA), 1X Mg^2+^-free DyNAzime EXT Buffer (FINNZYMES OY, Finland), 8% DMSO (FINNZYMES OY, Finland), 2.5 mM MgCl_2_ (FINNZYMES OY, Finland), 1 μM of GGC or CAG primers and 0.6 U of DyNAzime EXT DNA polymerase (FINNZYMES OY, Finland). PCR conditions for CAG were: 35 cycles of 95°C for 45 sec, 60°C for 45 sec, and 72°C for 1 min. PCR conditions for CGC were: 30 cycles of 95°C for 1 min, 55°C for 2 min, and 72°C for 2 min. PCR for CAG and GGC were initiated with a denaturation step of 95°C for 5 min, and terminated with an extension step at 72°C for 10 or 5 min, respectively.

PCR fragments separation of PCR product were performed by automated capillary electrophoresis, using an ABI PRISM 310 Genetic Analyzer (Applied Biosystems), and the length was determined with Genemapper Analysis Software (version V.3.2) (Applied Biosystems). 2 μL of the PCR product was mixed with 10 μL of formamide and 0.3 μL of GeneScan ROX-500 Size Standard (Applied Biosystems, USA CA), denatured at 98°C for 5 min. and cooled on ice.

The fragments size were compared in each capillary electrophoresis with PCR products obtained from men whose repeat lengths were known (standard automated sequencing, Macrogen Inc, Korea) in a single and pooled forms for CAG (13, 14, 17, 18, 19, 21, 22, 23, 24, 25, 28) and GGC (9, 13, 14, 17, 18). Standard automated sequencing was performed using two different amplicons that contained CAG or GGC repeats. The CAG and GGC amplicons were obtained after PCR reactions with CAG (A0 and A5) and GGC pair of primers (A3n and A10) as previously described [22]. Amplification was performed in a 25 L reaction volume, containing 150 ng of DNA, 200 M of each deoxynucleotide triphosphate (Invitrogen, USA), 1X Optimized DyNAzime EXT Buffer (FINNZYMES OY, Finland), 8% DMSO (FINNZYMES OY, Finland), 0.3 μM of each one sense or antisense primer and 1 U of DyNAzime EXT DNA polymerase (FINNZYMES OY, Finland). Both PCR reactions were performed under the same conditions: 37 cycles of 94°C for 1 min, 58°C for 1 min and 72°C for 1 min; initiated with a denaturation step of 94°C for 3 min, and terminated with an extension step at 72°C for 10 min.

The protocol was approved by the Institutional Review Boards of the Hospital San Borja Arriaran and the Faculty of Medicine, University of Chile. All parents signed an informed consent at the beginning of the study.

### Statistical analysis

Results are expressed as mean ± SD. Statistical analysis was performed using SPSS 10.0 for Windows (SPSS Inc., Chicago, IL). Normality of variables was assessed using the Kolmogorov-Smirnov test. Differences between girls with hypertrichosis and control girls were assessed by the Kruskall-Wallis or the Mann–Whitney test for non-parametric variables. To compare the number of CAG and GGC repeats in short, large and total alleles between girls with hipertrichosis and control girls we used the chi-square test. *P* < 0.05 was considered statistically significant.

## Results

### Hypertrichosis score and general assessment

The mean hypertrichosis score in the control group was 3 ± 2 and, as expected, none of these girls expressed concern about their body hair. The mean score observed in the girls with hypertrichosis was 16 ± 7, which was significantly higher compared to the control group (Table 1). The girls with hypertrichosis had long and dense vellous hair covering mainly the trunk, sacrum, upper and lower limbs, exceeding what is commonly observed in our population of prepubertal girls.

**Table 1:** Baseline characteristics of cases and controls

	**Cases**	**Controls**
Age (yrs)	6 ± 2	6 ± 2
Bone Age (yrs)	5.5 ± 0.3	6.7 ± 0.4
Hypertrichosis score	**16 ± 4**	3 ± 2**
BMI (k/m^2^) SDS	1 ± 0.1	0.9 ± 0.3
Follicle number per ovary	4.8 ± 2.4	3.7 ± 1.3

mean ± SD; Kruskall-Wallis test **P < 0.01 cases vs controls.

The mean chronological and bone age was similar in the girls with hypertrichosis and the control group, as shown in Table 1. Their weight and BMI were also comparable (Table 1).

### Ultrasound study

We did not find any differences in uterine or ovarian size between both groups of girls. The number of follicles per ovary, however, was higher in girls with hypertrichosis compared with controls (4.8 ± 0.5 vs 3.7 ± 0.3, p < 0.05) (Table 1).

### Hormone assays

Basal serum levels of LH, FSH and estradiol levels were similar in both groups of girls, and in the range usually observed in prepubertal girls. Basal DHEA-S, 17OH progesterone, androstenedione, inhibin-B and AMH were similar in both groups, and 17 OH progesterone and androstenedione. Interestingly, girls with hypertrichosis had significantly lower levels of SHBG than control girls (71.16 ± 16 vs 82 ± 15, p < 0.05). We did not observe statistically significant differences in the free androgen index between both groups of girls (0.83 ± 0.67 vs 0.62 ± 0.36 in hypertrichosis vs controls, p = NS) (Table 2).

**Table 2:** Hormonal profile of cases and controls

	**Cases**	**Controls**
Testosterone (ng/mL)	0.14 ± 0.06	0.14 ± 0.06
Androstenedione (ng/mL)	0.3 ± 0.4	0.2 ± 0.2
DHEAS (ng/mL)	116 ± 162	96 ± 84
**SHBG (nmol/L)**	**71 ± 16**	82 ± 15*
Free androgen index	0.83 ± 0.67	0.62 ± 0.36
Estradiol (pg/mL)	7.85 ± 5.0	8.2 ± 4.5
Inhibin (pg/mL)	16 ± 14	11 ± 4
AMH(pM)	23.4 ± 2.0	29.6 ± 4.2
Insulin (uUI/ml)	5.9 ± 0.6	5.6 ± 0.1

mean ± SD; Kruskall-Wallis test *P < 0.05 cases vs controls.

### CAG repeats analysis

The mean number of CAG repeats was 17.5 ± 3.1 vs 17.4 ± 3.0 in girls with hypertrichosis and control girls respectively, which was within the normal range observed in the general population [23,24]. Allele distribution for CAG repeats length was homozygous in 5 and 7% of cases and control girls respectively, and heterozygous in 95% and 93% of cases and control girls respectively, not showing any differences between cases and controls.

### GGC repeat analysis

The mean number of GGC repeats was not statistically significantly different between both groups (17.4 ± 1.1 vs 17.4 ± 0.61 in cases and controls respectively), and remained within the normal range for the general population [23,24]. Examination of the allele distribution did not reveal any difference in alleles with 17 or less repeats in the girls with hypertrichosis compared with controls. The GGC repeats length was homozygous in 70% and 69% of cases and controls, respectively, and heterozygous in 30% and 31% of cases and controls, respectively.

The combined analysis of CAG/GGC, however, showed a significantly higher prevalence of the most androgen-sensitive haplotypes (1–2: <22CAG + 17/17GGC - <14CAG + 17/18GGC) in the girls with hypertrichosis compared with controls (36% vs 14%, p < 0.05). Distribution of combination CAG/GGC haplotypes is shown in Table 3. In girls with the most androgen-sensitive haplotypes testosterone, DHEAS and androstenedione levels were significantly lower than in the girls with less sensitive haplotypes (p < 0.03).

**Table 3:** Distribution of combination CAG/GGC haplotypes

**Hap N°**	**CAG/GGC haplotypes definition**	**Girls**		**Total**
		**Hypertrichosis**	**Controls**	**(n)**
1	<22CAG + 17/17GGC	12 (28.6%)	4 (13.8%)	16
2	<14CAG + 17/18GGC	3 (7.1%)	0 (0%)	3
3	(≥18 and < 22)CAG + 17/18GGC	3 (7.1%)	6 (20.7%)	9
4	<22CAG + (<17 and/or >17)GGC	5 (11.9%)	6 (20.7%)	11
5	≥22/22CAG + (17 and/or 18)GGC	18 (42.9%)	13 (44.8%)	31
	Total (n)	41	29	70

One girl with hypertrichosis had a genotype: 20/27 CAG and 14/17 GGC and therefore did not classify in any of this haplotypes.

The prevalence of the most sensitive combinations (1 and 2) was significantly higher in girls with hypertrichosis than in controls (37.5% vs 13.8% P = 0.04 Chi^2^ test).

The hormonal levels according to CAG/GGC haplotypes are shown in Table 4. We observed that girls with hypertrichosis who harbor haplotypes 3–5 have lower SHBG concentrations compared to controls with the same haplotype.

**Table 4:** Hormonal levels according to CAG/GGC haplotypes

	**Cases (n)**		**Controls (n)**	
	**Hap 1-2**	**Hap 3-5**	**Hap 1-2**	**Hap 3-6**
Age (years)	5.9 ± 2.2 (13)	59 ± 1.(23)	7.0 ± 1 8 (4)	6.0 ± 1.5 (25)
Hypertrichosis score	16.0 ± 3.3 (13)&	17.1 ± 3.2 (23)&	1.8 ± 1.0 (4)	3.5 ± 2.0 (25)
Follicle number per ovary	4.9 ± 1.5 (9)	5.0 ± 3.0 (17)	3.3 ± 1.0 (3)	3.8 ± 1.4 (15)
LH (mUI/mL)	0.5 ± 0.4 (11)	0.4 ± 0.1 (19)&	0.5 ± 0.1 (4)	0.5 ± 0.2 (22)
FSH	2,0 ± 1.0 (11)	2.0 ± 1.1 (19)	2.4 ± 0.8 (4)	2.9 ± 3.2 (22)
Testosterone (ng/mL)	0.1 ± 0.06 (11)*	0.2 ± 0.06 (23)	02 ± 0.08 (4)	0.1 ± 0.06 (23)
Androstenedione (ng/mL)	0.2 ± 0.6 (11)*	0.4 ± 0.49 (20)	0.2 ± 0.08 (4)	0.2 ± 0.16 (22)
DHEAS (ng/mL)	38 ± 39 (11)*	161 ± 190 (22)	81 ± 82 (4)	98 ± 86 (23)
17OH progesterone (ng/mL)	0.5 ± 0.2 (12)	0.7 ±0.4 (20)	04 ± 0.15 (4)*	0.8 ± 0.5 (22)
Estradiol	8.74 ± 6.5 (11)	749 ± 4.2 (19)	7.45 ± 3.7 (4)	8.38 ± 4.7 (22)
SHBG (nmoL/L)	77.2 ± 16.7 (12)	68.2 ± 15.6 (19)&	79.3 ± 18 (4)	82.4 ± 15 (22)
FAI	0.54 ± 0.36 (11)	0.98 ± 0.77 (22)&	0.64 ± 0.18 (41)	0.62 ± 0.39 (23)
AMH (pmol/L)	41.6 ± 24.7 (12)*	23.8 ± 17.9 (20)	30.1 ± 5.9 (4)	22.4 ± 10.2 (22)
Inhibin B (pg/mL)	18.9±18 (12)&	14 ± 11 (20)	8.3 ± 3.3 (4)	12 ± 4.1 (22)

Values are mean **±** DS. *P<0.03 hap 1–2 versus hap 3–6 in cases or controls; &P<0.05 cases vs controls. Krustal Wallis Test.

In contrast, we observed that cases with the haplotypes 1–2 have normal androgen levels.

## Discussion

Prepubertal hypertrichosis is a poorly understood clinical entity which may cause significant patient and parental anxiety. In this study, we performed an assessment of ovarian function and androgen receptor repeat polymorphisms in a large group of carefully selected prepubertal girls with this condition, which were compared with a group of control girls.

The limited data available regarding the androgen profile of girls with prepubertal hypertrichosis are somewhat controversial. Balducci and Toscano demonstrated elevated concentrations of serum dihydrotestosterone (DHT) in a small group of prepubertal girls with hypertrichosis compared with age-matched controls. These authors, however, did not observe a concomitant increase in the levels of the DHT metabolite 3α- androstanediol glucuronide [12]. Gryngarten et al. observed increased serum testosterone levels and FAI in a group of prepubertal girls with hypertricosis compared to controls. In addition, they observed a slight increase in 3α-androstanediol glucuronide concentrations in approximately half of their girls with hypertrichosis [13].

We evaluated 42 prepubertal girls with hypertrichosis that were matched with 29 control prepubertal girls. We did not observe any differences in the androgen profile of both groups, except for lower levels of SHBG in the girls with hypertrichosis, although FAI was not different. In addition, serum AMH levels and pelvic ultrasound were similar in both groups. We also performed an hormonal study in both groups of girls, but we did not observe any differences in the basal concentrations of gonadotropins or sex steroid concentrations.

The investigation of the androgen receptor repeat polymorphisms provided the most interesting results of this study. Androgen receptor CAG repeats usually range between 11 and 35, and a decreased CAG repeat number has been linked to an increased transcriptional response to androgens [25]. Van Nieuwerburgh et al. studied 97 oligo-anovulatory women with ultrasound features of PCOS, and observed that patients with a bi-allelic mean lower than 21 repeats had lower androgen levels, but more florid clinical evidence of acne and/or hirsutism [26]. Ibañez et al. observed that girls with precocious pubarche had shorter mean CAG repeats and a greater proportion of short alleles (20 repeats or less) compared to controls. They concluded that shorter androgen receptor CAG number is indicative of increased androgen sensitivity, and subsequent ovarian hyperandrogenism [27]. In addition, Vottero et al. reported a reduced androgen receptor gene methylation pattern, which was associated with the presence of shorter CAG repeats, in girls with precocious pubarche. This constellation of findings might lead to hypersensitivity of the hair follicles to androgens, and therefore to the premature development of pubic hair [28].

On the other hand, *in vitro* characterization has showed a higher transactivating capacity for GGN 23 allele (GGC 17), and GGN 27 or GGN 10, compared to GGN 24 with a constant CAG repeat number of CAG 22, in response to testosterone analogs (R1881) and 5-α dihydrotestosterone. In accordance with several reports, our GGC distribution showed that GGC 17 and GGC 18 were the most frequent alleles in our population.

In the present study we did not find differences in the mean number of CAG or GGC repeats. In order to investigate the combined contribution of the CAG and GGC alleles to androgen sensitivity, we performed a study of the joint distribution of these alleles. The combined analysis of CAG/GGC showed a significantly higher prevalence of the most androgen-sensitive combinations (<18 CAG + 17/17 GGC and <14 CAG + 17/18 GGC) in the girls with hypertrychosis. The normal androgen levels in haplotypes 1 and 2 indicates that they may develop hypertrichosis due to enhanced androgen receptor sensitivity. The higher AMH and inhibin B levels observed in these patients, suggests that they appear to have a higher number of small antral follicles, as observed in patients with PCO. In addition, the girls who harbored combinations 3 – 5 had lower SHBG concentrations compared to controls. This hormonal pattern may lead to the development of hypertrichosis due to a higher free androgen index. The lower LH levels observed in these patients may be consequence of the central negative feedback by androgens.

Although it is known that analyzing blood DNA may not reflect target tissue AR sensitivity, we did not perform skin biopsies to study AR sensitivity due to ethical considerations.

## Conclusions

In conclusion, we have studied the hormonal profile and androgen receptor polymorphisms in a group of prepubertal girls with hypertrichosis. Girls with hypertrichosis exhibited lower levels of SHBG, but had otherwise normal androgen levels. The association of GGC 17 + CAG < 18 repeats suggests that some girls with hypertrichosis may have androgen receptors with enhanced sensitivity, which may facilitate the growth of their body hair. In order to clarify the contribution of each androgen receptor repeat, it will be important to study the methylation of the androgen receptor in these patients.

## Competing interests

The authors declare that they have no competing interests.

## Authors’ contributions

MIH: Conceived the study, participated in the design of the study, recruited the patients and controls, performed the physical examination, performed the statistical analysis and drafted the manuscript. AC: Participated in the determination of CAG and GGC repeats, molecular analysis and statistical analysis. KB: Participated in the determination of CAG and GGC repeats and molecular analysis. AA: Nurse who drew the blood samples. AE: Participated evaluating the bone age. LT: Participated performing the Ultrasound. GI: Participated carried out the immunoassays and statistical analysis. EC: Participated in the design of the study. FC: Participated in the design of the study and helped to draft the manuscript. All the authors read an approved the final manuscript.

## References

[B1] Beighton P Familial hypertrichosis cubiti: hairy elbows syndrome J Med Genet 1970 7 158 160 10.1136/jmg.7.2.158 5519603 PMC1468803

[B2] Barth JH Wilkinson JD Dawber RP Prepubertal hypertrichosis: normal or abnormal? Arch Dis Child 1988 63 666 668 10.1136/adc.63.6.666 3389902 PMC1778859

[B3] Baumeister FA Egger J Schildhauer MT Stengel-Rutkowski S Ambras syndrome: delineation of a unique hypertrichosis universalis congenita and association with a balanced pericentric inversion(8)(p11.2; q22) Clin Genet 1993 44 121 128 8275569 10.1111/j.1399-0004.1993.tb03862.x

[B4] Baumeister FA Differentiation of Ambras syndrome from Hypertrichosis Universalis Clin Genet 2000 57 157 158 10735640 10.1034/j.1399-0004.2000.570213.x

[B5] Beighton P Congenital hypertrichosis lanuginosa Arch Dermatol 1970 101 669 672 10.1001/archderm.1970.04000060041010 5424483

[B6] Trüeb RM Causes and management of hypertrichosis Am J Clin Dermatol 2002 3 617 627 10.2165/00128071-200203090-00004 12444804

[B7] Hassan G Khalaf H Mourad W Dermatologic complications after liver transplantation: a single-center experience Transplant Proc 2007 39 1190 4194 10.1016/j.transproceed.2007.04.009 17524929

[B8] Hengge UR Ruzicka T Schwartz RA Cork MJ Adverse effects of topical glucocorticosteroids J Am Acad Dermatol 2006 54 1 15 10.1016/j.jaad.2005.01.010 16384751

[B9] Mihatsch MJ Kyo M Morozumi K Yamaguchi Y Nickeleit V Ryffel B The side-effects of ciclosporine-A and tacrolimus Clin Nephrol 1998 49 356 363 9696431

[B10] Tosi A Misciali C Piraccini BM Peluso AM Bardazzi F Drug-induced hair loss and hair growth. Incidence, management and avoidance Drug Saf 1994 10 310 317 10.2165/00002018-199410040-00005 8018303

[B11] Vanderveen EE Ellis CN Kang S Case P Headington JT Voorhees JJ Swanson NA Topical minoxidil for hair regrowth J Am Acad Dermatol 1984 11 416 421 10.1016/S0190-9622(84)70183-6 6384289

[B12] Balducci R Toscano V Bioactive and peripheral androgens in prepubertal simple hypertrichosis Clin Endocrinol (Oxf) 1990 33 407 414 10.1111/j.1365-2265.1990.tb00506.x 2147601

[B13] Gryngarten M Bedecarràs P Ayuso S Bergadà C Campo S Escobar ME Clinical assessment and serum hormonal profile in prepubertal hypertrichosis Horm Res 2000 54 20 25 10.1159/000063432 11182631

[B14] Eder IE Culig Z Putz T Nessler-Menardi C Bartsch G Klocker H Molecular biology of the androgen receptor: from molecular understanding to the clinic Eur Urol 2001 40 241 251 11684838 10.1159/000049782

[B15] Mangelsdorf DJ Thummel C Beato M Herrlich P Schütz G Umesono K Blumberg B Kastner P Mark M Chambon P Evans RM The nuclear receptor superfamily: the second decade Cell 1995 83 835 839 10.1016/0092-8674(95)90199-X 8521507 PMC6159888

[B16] Lundin KB Giwercman A Dizeyi N Giwercman YL Functional in vitro characterisation of the androgen receptor GGN polymorphism Mol Cell Endocrinol 2007 264 184 187 10.1016/j.mce.2006.11.008 17197074

[B17] Greulich WW Pyle SI Radiographics atlas of skeletal development of the hand and wrist 1959 2 Standford (CA): Standford Universtity Press

[B18] Swanson M Sauerbrei EE Cooperberg PL Medical implications of ultrasonically detected polycystic ovaries J Clin Ultrasound 1981 9 219 222 10.1002/jcu.1870090504 6787087

[B19] Vermeulen A Verdonck L Kaufman JM A critical evaluation of simple methods for the estimation of free testosterone in serum J Clin Endocrinol Metab 1999 84 3666 3672 10.1210/jcem.84.10.6079 10523012

[B20] Edwards SM Badzioch MD Minter R Hamoudi R Collins N Ardern-Jones A Dowe A Osborne S Kelly J Shearer R Easton DF Saunders GF Dearnaley DP Eeles RA Androgen receptor polymorphisms: association with prostate cancer risk, relapse and overall survival Int J Cancer 1999 84 458 465 10.1002/(SICI)1097-0215(19991022)84:5<458::AID-IJC2>3.0.CO;2-Y 10502720

[B21] Vottero A Stratakis CA Ghizzoni L Longui CA Karl M Chrousos GP Androgen receptor-mediated hypersensitivity to androgens in women with nonhyperandrogenic hirsutism: skewing of X-chromosome inactivation J Clin Endocrinol Metab 1999 84 1091 1095 10084600 10.1210/jcem.84.3.5540

[B22] Ferlin A Garolla A Bettella A Bartoloni L Vinanzi C Roverato A Foresta C Androgen receptor gene CAG and GGC repeat lengths in cryptorchidism Eur J Endocrinol 2005 152 419 425 10.1530/eje.1.01860 15757859

[B23] Esteban E Rodon N Via M Gonzalez-Perez E Santamaria J Dugoujon JM Chennawi FE Melhaoui M Cherkaoui M Vona G Harich N Moral P Androgen receptor CAG and GGC polymorphisms in Mediterraneans: repeat dynamics and population relationships J Hum Genet 2006 51 129 136 10.1007/s10038-005-0336-7 16365681

[B24] Kittles RA Young D Weinrich S Hudson J Argyropoulos G Ukoli F Adams-Campbell L Dunston GM Extent of linkage disequilibrium between the androgen receptor gene CAG and GGC repeats in human populations: implications for prostate cancer risk Hum Genet 2001 109 253 261 10.1007/s004390100576 11702204

[B25] Chamberlain NL Driver ED Miesfeld RL The length and location of CAG trinucleotide repeats in the androgen receptor N-terminal domain affect transactivation function Nucleic Acids Res 1994 22 3181 3186 10.1093/nar/22.15.3181 8065934 PMC310294

[B26] Van Nieuwerburgh F Stoop D Cabri P Dhont M Deforce D De Sutter P Shorter CAG repeats in the androgen receptor gene may enhance hyperandrogenicity in polycystic ovary syndrome Gynecol Endocrinol 2008 24 669 673 10.1080/09513590802342841 19172534

[B27] Ibáñez L Ong KK Mongan N Jääskeläinen J Marcos MV Hughes IA De Zegher F Dunger DB Androgen receptor gene CAG repeat polymorphism in the development of ovarian hyperandrogenism J Clin Endocrinol Metab 2003 88 3333 3338 10.1210/jc.2002-021791 12843184

[B28] Vottero A Capelletti M Giuliodori S Viani I Ziveri M Neri TM Bernasconi S Ghizzoni L Decreased androgen receptor gene methylation in premature pubarche: a novel pathogenetic mechanism? J Clin Endocrinol Metab 2006 91 968 972 10.1210/jc.2005-2354 16403814

